# Integrating emergency medical services and hospital surge capacity for mass-gathering preparedness: a structured narrative review

**DOI:** 10.3389/fmed.2026.1861093

**Published:** 2026-09-28

**Authors:** Abdullah Basnawi

**Affiliations:** Faculty of Medicine, Department of internal medicine, University of Tabuk, Tabuk, Saudi Arabia

**Keywords:** disaster preparedness, emergency medical services, hospital surge, mass gathering, patient presentation rate, prehospital care, surge capacity

## Abstract

**Background:**

Mass gatherings can generate concentrated, time-dependent demand across in-event health services, municipal emergency medical services (EMS), emergency departments, and inpatient services. Evidence relevant to preparedness is heterogeneous and is often reported with inconsistent denominators, limiting direct comparison.

**Methods:**

This structured narrative review synthesizes published evidence on EMS and hospital surge-capacity integration across the four surge domains of staff, stuff/supplies, space, and systems. The review retained the original structured search of MEDLINE/PubMed, Embase, Scopus, Web of Science, and the Cochrane Library for 2010-February 2026 and incorporated a focused PubMed and citation update through September 2026. Because the purpose is interpretive synthesis rather than exhaustive effect estimation, no *de novo* meta-analysis or pooled estimates were produced; quantitative findings are reported with the denominators and analytic context used by the source publications.

**Results:**

A 2025 scoping review of 41 papers reported medical-doctor presence in 34/41 papers (82.9%), medication interventions in 23/41 (56.1%), procedural interventions in 23/41 (56.1%), health-support systems in 18/41 (43.9%), mobile treatment spaces in 23/41 (56.1%), and temporary structures in 21/41 (51.2%). That review reported median calculated patient-presentation and transport-to-hospital rates of 6.2 and 0.35 per 1,000 attendees, respectively, whereas a separate 22-article stadium review reported much lower medians when standardized per 10,000 spectators. Hospital effects were directionally heterogeneous: a 14-study systematic review found event-associated emergency-department changes ranging from 7.5%–37% decreases to increases as high as 83% in specific settings. Digital integration has shown promising simulated gains, but clinical effectiveness remains insufficiently validated.

**Conclusion:**

Preparedness is best treated as an integrated continuum rather than as separate event-site and hospital plans. Standardized denominators, linked patient-level data, context-specific resource design, and prospective evaluation are priorities.

## 1 Introduction

Mass-gathering medicine spans planned sporting and cultural events, music festivals, religious pilgrimages, and spontaneous gatherings. The contemporary evidence base is large but uneven: a recent bibliometric review identified 898 relevant publications after screening, while also showing important imbalances in event types and research topics (1). Conceptual work has long emphasized that the health consequences of an event depend not only on crowd size but also on environmental exposure, event duration, population characteristics, venue design, behavior, and the configuration of available health services (2).

For preparedness purposes, surge capacity is commonly organized around four interacting domains: staff, stuff/supplies, space, and systems. These domains should not be interpreted as independent inventories. Their operational value depends on coordination, communications, command arrangements, transport pathways, and the receiving capacity of the wider health system. Flexible-surge models similarly emphasize collaboration across organizational boundaries when routine or preplanned capacity is insufficient (3, 4).

EMS and in-event health services form the first clinical interface, but they are only one part of the care continuum. An earlier systematic review of 24 mass-gathering studies found that 23 described in-event first-responder services, all described ambulance transport, yet only nine reported patients presenting to hospital; the authors therefore called for linked patient-level data across event, ambulance, and hospital settings (5). Standardized reporting initiatives have also recommended consistent event and clinical variables to improve comparability across studies (6).

More recent evidence illustrates both progress and persistent heterogeneity. A 2025 scoping review of 41 papers catalogued in-event interventions across the four surge domains and reported calculated patient-presentation and transport-to-hospital benchmarks (7). A stadium-specific scoping review of 22 articles found markedly different rates when outcomes were standardized per 10,000 spectators (8). At the system level, register data from nine Finnish mass gatherings demonstrated increased EMS and rescue workloads around most events (9), while a 14-study systematic review found that emergency-department (ED) attendance may decrease during some events but increase substantially during others (10). These findings argue against a single universal surge multiplier.

Clinical and operational context further modifies demand. A retrospective study of a large electronic-dance-music festival found intoxication and poisoning to be a major driver of acute-care presentations and reported that enhanced on-site care was associated with lower ambulance transfer rates without changing the overall transfer-to-hospital rate (11). A digital emergency-information platform spanning on-site teams, ambulances, and hospitals demonstrated promising simulation-based gains in survival and evacuation efficiency, but those results are not equivalent to proven patient benefit in routine practice (12). Spontaneous gatherings, Hajj, and geographically remote events create additional requirements for security, language support, route management, self-sufficiency, and interagency coordination (13–15). Infectious-disease and antimicrobial-resistance risks at religious mass gatherings also illustrate how hospital preparedness may extend beyond immediate trauma and acute illness (16).

### 1.1 Aim and objectives

This structured narrative review aims to critically synthesize how EMS, in-event health services, and hospital surge capacity can be integrated for mass-gathering preparedness. The objectives are to: (i) clarify the role of the four surge-capacity domains; (ii) summarize published PPR, TTHR, EMS-workload, and ED-impact evidence using explicit denominators; (iii) examine operational and digital mechanisms linking prehospital and hospital care; (iv) identify context-specific requirements for planned, spontaneous, religious, and remote gatherings; and (v) identify methodological limitations and priorities for research and practice.

## 2 Research methods

### 2.1 Study design and rationale

This article is a structured narrative review, not a scoping review or systematic review. A narrative approach was retained because the review question is integrative and spans heterogeneous evidence types, including systematic and scoping reviews, retrospective observational studies, qualitative studies, conceptual surge-capacity frameworks, and implementation/simulation research. The manuscript was revised in accordance with the Scale for the Assessment of Narrative Review Articles (SANRA) to make the review question, search approach, referencing, scientific reasoning, and presentation of relevant endpoint data explicit (17). No PRISMA claim is made, and the manuscript does not present the source-selection process as if it were a prospectively registered systematic review.

### 2.2 Search strategy and information sources

The original search was conducted between January and February 2026 across MEDLINE (via PubMed), Embase, Scopus, Web of Science Core Collection, and the Cochrane Library for English-language publications from January 2010 through February 2026. Search concepts combined terms for mass gatherings (including mass gathering medicine, crowd medicine, sporting events, music festivals, pilgrimages, and spontaneous gatherings) with terms for emergency medical services, prehospital care, ambulance services, emergency departments, hospital surge capacity, disaster preparedness, patient presentation rate, and transport-to-hospital rate. Reference lists of key reviews and relevant specialist papers were also checked. For the present revision, a focused PubMed and citation update was undertaken through September 2026 to identify newly published peer-reviewed evidence directly relevant to EMS delivery, hospital impact, and prehospital-hospital integration; this update identified, among other relevant material, a 2026 qualitative study of EMS delivery during Hajj (14).

### 2.3 Eligibility and evidence prioritization

Evidence was considered when it addressed a mass-gathering setting and contributed directly to at least one of the following: in-event clinical capability, EMS workload or transport, ED or hospital demand, surge-capacity design, prehospital-hospital information exchange, or operational coordination. Eligible evidence included peer-reviewed published or accepted original research, systematic/scoping/integrative reviews, and relevant conceptual or consensus work. Simulation studies were retained only when they addressed the prehospital-hospital interface and are explicitly identified as simulation evidence. Preprints, unpublished original data, conference-only evidence without a full peer-reviewed article, duplicate publications, and papers in which the mass-gathering context was peripheral were excluded or de-emphasized. Infectious-disease studies were considered only when they had a clear implication for acute-care or surge planning.

### 2.4 Evidence selection and review workflow

Because this is a single-author narrative review, the author performed relevance selection and synthesis; the manuscript no longer claims independent duplicate screening, formal dual-reviewer extraction, or design-specific quality scoring. Those procedures would imply a systematic-review architecture that was not the intended design. Instead, higher-level peer-reviewed reviews were prioritized for broad quantitative summaries, and representative primary studies were used to illustrate operational mechanisms or contexts not adequately captured by secondary evidence. To avoid double counting, results already synthesized within a cited review were not re-pooled with individual primary studies from that same evidence base. Figure 1 summarizes the transparent narrative source-selection and synthesis workflow. It is intentionally not labeled as a PRISMA diagram because exact record-level screening counts were not prospectively maintained for this narrative review.

**Figure 1:**
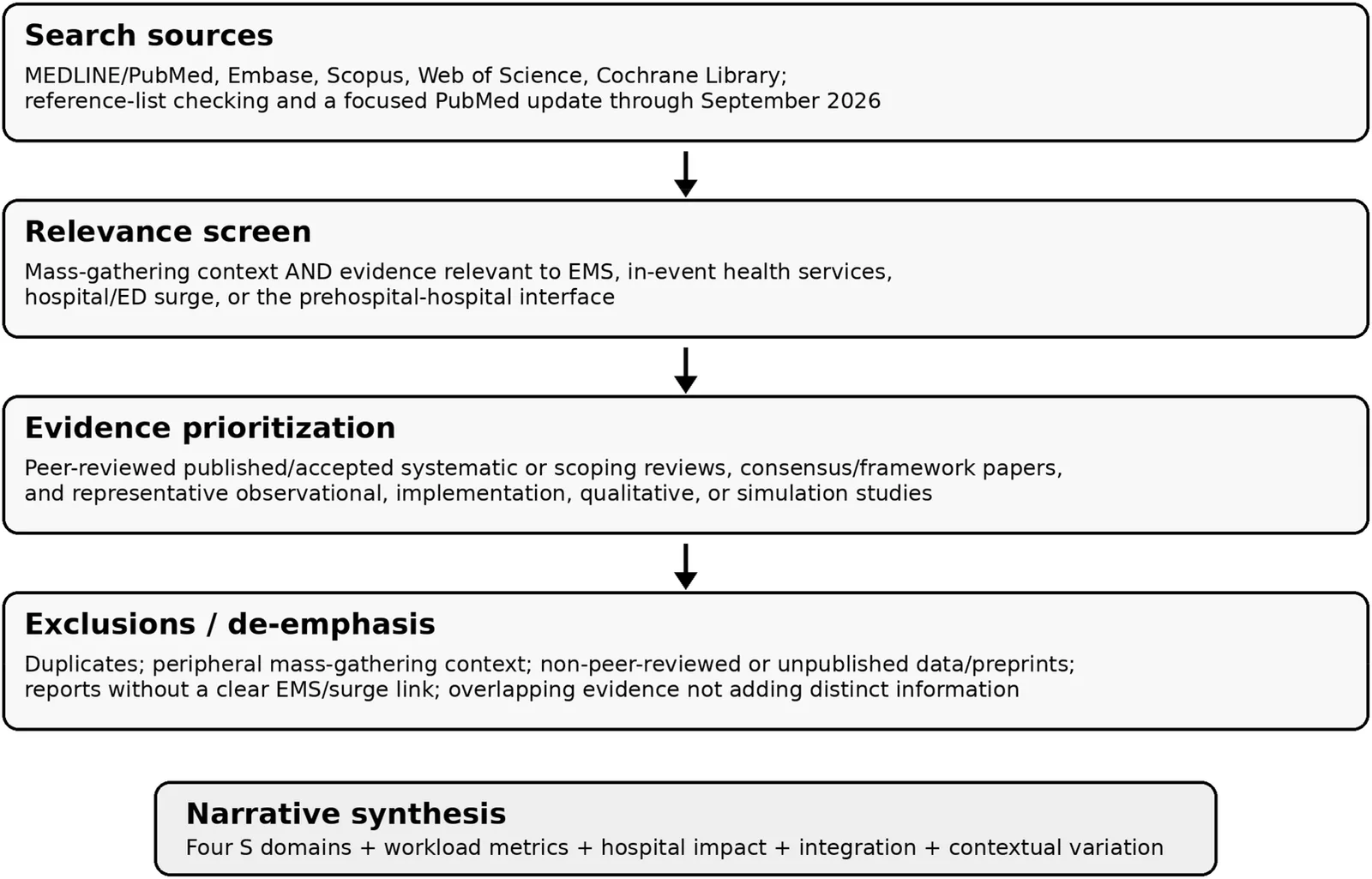
Structured narrative review evidence-selection and synthesis workflow.

### 2.5 Data extraction, critical interpretation, and synthesis

For each source used to support quantitative or operational conclusions, the review extracted or verified the study/review design, event setting, sample or study denominator where reported, outcome definition, rate denominator, principal findings, and important limitations. Percentages are accompanied by absolute n/N values when these are reported by the source and are meaningful. Rate denominators are retained exactly (for example, per 1,000 attendees versus per 10,000 spectators) rather than silently converted across heterogeneous settings.

No *de novo* statistical pooling or meta-analysis was undertaken. In particular, the median PPR and TTHR values presented below are published summary estimates from the 41-paper scoping review by Ranse and colleagues (7), not pooled estimates newly calculated for the present article. Similarly, stadium-specific medians are reproduced from Sultana and colleagues (8). The synthesis is therefore interpretive: findings are organized across the four surge domains and the prehospital-hospital continuum, with observational associations separated from simulation outputs and with causal language avoided unless supported by the underlying design.

Use of generative AI during revision. ChatGPT (OpenAI; GPT-5.6 Sol) was used during revision for language editing, structural refinement, and assistance with citation cross-checking. Generative AI was not treated as an author or an evidence source. Scientific interpretation, decisions about inclusion, and responsibility for the accuracy of the final manuscript remain with the author.

## 3 Results

### 3.1 Evidence base and reporting conventions

The evidence relevant to EMS-hospital integration is heterogeneous in design, event type, geography, outcome definitions, and denominator conventions. Accordingly, this review does not report a single number of “included studies” as if all sources formed one new pooled cohort. Instead, source-level study counts are reported where they belong. For example, Ranse et al. synthesized 41 papers on in-event health interventions (7), Sultana et al. synthesized 22 stadium/arena papers (8), PoshtMashhadi et al. synthesized 14 studies of ED impact (10), and Ranse et al. synthesized 10 papers on spontaneous and unplanned gatherings (13). This distinction is central to interpreting the strength and generalizability of the quantitative findings.

### 3.2 Surge-capacity domains: staff, stuff/supplies, space, and systems

The most useful quantitative description of reported in-event interventions comes from the 41-paper scoping review by Ranse et al. (7). Table 1 reproduces its principal counts with absolute numerators and denominators. These values indicate how frequently an intervention type was reported in the included papers; they do not establish that the intervention is effective, necessary at every event, or superior to an alternative model.

**Table 1:** Published counts of in-event health interventions across the four surge-capacity domains.

Domain	Intervention reported in the 41-paper scoping review	Absolute n/N	Percentage	Interpretive note	Source
Staff	Medical doctors present	34/41	82.9%	Frequency of reporting; not comparative effectiveness	(7)
Stuff/supplies	Medications	23/41	56.1%	Examples varied by event and scope of practice	(7)
Stuff/supplies	Procedural interventions	23/41	56.1%	Procedures ranged from basic to advanced on-site care	(7)
Space	Mobile treatment structures	23/41	56.1%	Reported configuration, not proof of ED avoidance	(7)
Space	Temporary structures	21/41	51.2%	Context and capacity varied substantially	(7)
Systems	Health-support systems	18/41	43.9%	Includes systems supporting delivery/coordination	(7)

#### 3.2.1 Staff

Medical doctors were reported in 34/41 papers (82.9%) in the Ranse review (7), making physician presence the most frequently reported staff-related intervention in that evidence set. This should not be interpreted as proof that physician-led models are universally superior. Staffing should instead be matched to event risk, scope of practice, expected case mix, transport times, and the availability of definitive care.

#### 3.2.2 Stuff/supplies

Medications were reported in 23/41 papers (56.1%), and procedural interventions were also reported in 23/41 (56.1%) (7). The practical implication is that on-site services often function beyond first aid, but the appropriate formulary and procedural capability remain context dependent. Music-festival settings, for example, may require specific toxicology capability, whereas prolonged outdoor or religious gatherings may require different hydration, respiratory, and chronic-disease resources.

#### 3.2.3 Space

Mobile treatment spaces were reported in 23/41 papers (56.1%) and temporary structures in 21/41 (51.2%) (7). Such spaces can support observation and treatment that might otherwise require transport, but the available literature does not justify a universal claim that temporary structures themselves reduce ED attendance. ED effects vary by event type and health-service model, as discussed in Section 3.3.

#### 3.2.4 Systems

Health-support systems were reported in 18/41 papers (43.9%) (7). The lower reporting frequency is notable because systems-level functions - communication, command, information sharing, transport coordination, patient tracking, and hospital interface - determine whether staff, supplies, and treatment space can be used coherently. Qualitative work with event managers, police, and medical personnel similarly identifies communication, risk ownership, and planning as recurring safety issues (18).

### 3.3 Patient-presentation rates, transport, EMS workload, and hospital impact

#### 3.3.1 Patient presentation and transport metrics

The 41-paper scoping review reported a median calculated PPR of 6.2 per 1,000 attendees (IQR 1.92–14.9) and a median calculated TTHR of 0.35 per 1,000 (IQR 0.05–0.61) (7). These are source-level descriptive benchmarks, not universal planning constants. In the stadium/arena review, 22 articles were included, of which 18/22 (81.8%) concerned sports and 3/22 (13.6%) music concerts. After the review authors standardized rates to 10,000 spectators, PPR ranged from 1 to 24 per 10,000 (median 3.8), and TTHR ranged from approximately 0.01 to 4 per 10,000 (median 0.35) (8). The marked difference from the broader 41-paper estimates underscores the importance of event setting, case definition, health-service configuration, and denominator. Table 2 summarizes the quantitative evidence and preserves the denominator used by each source.

**Table 2:** Quantitative evidence relevant to EMS workload, patient presentation, transport, and emergency-department impact.

Evidence source	Absolute study/sample context	Published quantitative finding	Denominator/caution
Ranse et al. scoping review (7)	41 papers	Median calculated PPR 6.2; IQR 1.92-14.9. Median calculated TTHR 0.35; IQR 0.05-0.61.	Rates per 1,000 attendees; descriptive summary from the source review, not re-pooled here.
Sultana et al. stadium/arena scoping review (8)	22 articles; sports 18/22 (81.8%); concerts 3/22 (13.6%)	PPR 1-24, median 3.8; TTHR approximately 0.01-4, median 0.35.	Rates standardized by source authors per 10,000 spectators; not directly interchangeable with all-event estimates.
Koski et al. register study (9)	25,124 EMS/rescue missions; 9 mass-gathering events plus reference periods	Missions increased around most events; violence-related missions RR 1.87 (95% CI 1.43-2.44).	System workload measure; not a patient-presentation rate.
PoshtMashhadi et al. systematic review (10)	14 studies	Some events showed 7.5%-37% reductions in ED attendance; other settings showed increases up to 83%.	Direction and magnitude varied by event, diagnosis, timing, and setting; not a single pooled effect.

### 3.4 Clinical case mix and host-system workload

Mass gatherings may alter both the volume and the composition of demand. In Finland, a retrospective register analysis covered 25,124 EMS and rescue missions across nine mass-gathering events and matched reference periods; overall workload increased around most events, and violence-related missions showed the largest relative increase (RR 1.87, 95% CI 1.43–2.44) (9). At a Calgary electronic-dance-music festival attracting approximately 35,000 attendees annually, a 2015–2019 retrospective study reported intoxication/poisoning in 63.9% of mass-gathering-related acute-care presentations and found lower ambulance-transfer rates after enhanced on-site care, without a corresponding reduction in the overall TTHR (11). The published abstract does not provide the absolute numerator and denominator for the 63.9% value; therefore, this review does not invent an n/N.

### 3.5 Hospital surge mechanisms and prehospital-hospital integration

Emergency-department effects are not uniformly upward. A 2025 systematic review of 14 studies found reductions in total ED attendance of approximately 7.5%–37% in some mass-gathering settings, but increases in other settings, reaching as high as 83% during Umrah; trauma and substance-related demand also varied by event type, and surges could occur several hours after the event (10). Thus, event-associated “surge” should be understood as a time- and diagnosis-dependent change in workload rather than an assumption of simple volume increase.

Enhanced on-site capability may divert or definitively treat selected low-acuity patients, but the evidence is predominantly observational and confounded by event risk, attendee behavior, access thresholds, staffing, local EMS practice, and hospital geography. The most defensible operational interpretation is that on-site care can influence transport pathways and case mix; whether it decreases net ED burden in a specific setting must be measured rather than assumed (10, 11).

Digital integration offers a potential systems-level mechanism. Tian et al. developed a unified emergency medical information system linking 17 on-site medical locations, five ambulances, and three hospitals, involving 39 medical staff (12). Their evacuation algorithm produced approximately a 5% improvement in simulated overall survival and a 50% improvement in average evacuation efficiency. These outcomes are encouraging but must be labeled correctly: the performance gains derive from simulation, not a randomized or controlled clinical evaluation. Real-world benefit will depend on interoperability, network reliability, user training, privacy/security, and local workflow integration. Table 3 summarizes this integration evidence alongside key context-specific evidence with absolute study, sample, or deployment information.

**Table 3:** Context-specific and integration evidence with absolute study/sample information.

Context/integration mechanism	Absolute evidence context	Main finding relevant to integration	Interpretive caution	Source
Digital information system	3 hospitals; 5 ambulances; 17 on-site medical locations; 39 medical staff	Simulation: approximately 5% higher overall survival and 50% higher average evacuation efficiency.	Simulation output; clinical effectiveness not established.	(12)
Spontaneous/unplanned gatherings	10 papers; riots/civil unrest 4/10 (40%); unplanned large parties 2/10 (20%)	EMS/pre-hospital personnel were predominant responders; some settings required ballistic/respiratory PPE and interagency planning.	Retrospective, heterogeneous event evidence.	(13)
Hajj EMS delivery	26 EMS-responder interviews+7 key-informant interviews; 49 codes identified	Traffic control, language barriers, missing medical history, non-emergency demand, and coordination affected EMS delivery.	Qualitative evidence from one Hajj context.	(14)
Remote sporting event	13 clinicians reported in the event medical team (6 attending physicians, 2 sports-medicine fellows, 3 emergency-medicine residents, 2 advanced practice providers)	Equipped on-site clinic illustrates greater self-sufficiency in a geographically isolated event.	Single-event descriptive report; no controlled effectiveness estimate.	(15)

### 3.6 Contextual variation

Spontaneous and unplanned gatherings differ fundamentally from planned events because there may be no opportunity to pre-position event-specific resources. A 2025 scoping review included 10 papers; four of 10 (40%) concerned riots/civil unrest and two of 10 (20%) unplanned large parties. EMS or prehospital personnel were the predominant health responders, and additional ballistic or respiratory protective equipment was identified in some settings, supporting a risk-based and interagency approach (13). Hajj introduces a different systems problem. A 2026 qualitative study used 26 semi-structured EMS-responder interviews plus seven key-informant interviews and identified traffic-control-point management, language barriers, incomplete medical histories, non-emergency demand, and interagency coordination as key operational issues (14). Remote events also require greater on-site self-sufficiency; a 2019 mountain-bike World Cup case report described a 13-clinician event medical team and an equipped on-site clinic in a geographically isolated setting (15). Finally, a systematic review of antimicrobial resistance screened 5,559 titles and included 44 studies, 40/44 of which involved religious gatherings; the authors could not meta-analyze because of heterogeneity but reported evidence of acquisition of resistant organisms after Hajj (16). These contexts demonstrate why staffing, supplies, space, and hospital interfaces cannot be specified by crowd size alone.

## 4 Discussion

The central finding of this structured narrative review is not a new pooled effect estimate. Rather, the evidence supports a systems interpretation of mass-gathering preparedness: in-event clinical capability, municipal EMS, transport, EDs, inpatient resources, and public-safety partners form a single operational continuum. The four surge domains provide a useful organizing framework, but “systems” is the enabling layer that connects the other three. Communication failure, delayed destination decisions, poor situational awareness, or unavailable receiving capacity can negate apparently adequate staffing, supplies, and treatment space.

The revised synthesis also changes how PPR and TTHR should be used. The 6.2/1,000 PPR and 0.35/1,000 TTHR medians are published descriptive summaries from a 41-paper scoping review, not values calculated by the present author (7). Stadium-specific medians are lower and are reported per 10,000 spectators (8). Both are useful for benchmarking within comparable settings, but neither should be transferred uncritically to a different event. Planning models should retain event-specific assumptions and clearly state the denominator, population definition, time window, and whether patients were treated on-site, transported by event EMS, self-presented, or arrived through routine ambulance channels.

The same caution applies to hospital impact. The 14-study review by PoshtMashhadi et al. found both decreases and increases in ED attendance (10). Reduced ED volume during an event may reflect delayed or displaced routine demand rather than a protective effect of event medical services, while post-event peaks can occur after the crowd disperses. Therefore, hospital preparedness should incorporate temporal surveillance and diagnostic case mix, not merely a predicted total number of presentations. Where possible, event planners and hospitals should predefine trigger thresholds for escalation and monitor real-time ED arrivals, ambulance availability, bed status, and transport destinations.

On-site treatment can be an important filtering layer, particularly for conditions that can be safely assessed, observed, and discharged without transport. However, the revised manuscript deliberately avoids stating that physician staffing or temporary field hospitals independently “reduce” hospital transfers unless the cited design supports that inference. The 41-paper scoping review primarily describes what interventions were reported, not comparative effectiveness (7). Similarly, the Calgary festival study suggests that stronger on-site capability can alter ambulance-transfer behavior, yet the overall TTHR did not change (11). These findings support tailored capability and prospective evaluation rather than a universal staffing prescription.

Digital information exchange is conceptually attractive because it addresses a recurring gap between prehospital and hospital services. The Tian platform demonstrates technical feasibility across multiple care locations and shows large simulated gains in evacuation efficiency (12). Nevertheless, simulation performance is vulnerable to assumptions about casualty distribution, transport times, bed availability, data latency, and algorithm compliance. Clinical deployment should therefore be evaluated using implementation outcomes such as time to definitive destination, secondary transfer, ED congestion, communication failures, data completeness, and patient-centered outcomes before claims of improved survival are generalized.

Context is equally important. Spontaneous gatherings may require responder protection and rapid interagency coordination without event-specific infrastructure (13). Hajj involves complex mobility, language, and multiagency constraints at an exceptional scale (14). Remote events may need greater procedural capability because transport distances are longer and backup resources fewer (15). Religious gatherings may also create infectious-disease and antimicrobial-resistance implications that persist beyond the event itself (16). The practical lesson is that a mass-gathering plan should be risk-based, not template-based.

A persistent weakness of the literature is limited linkage across the full patient journey. The 2017 systematic review found that only nine of 24 included studies reported hospital presentations despite all reporting ambulance transport (5). More recent studies have improved event-level reporting, but denominators and follow-up remain inconsistent. The minimum-data-set work in mass-gathering research remains relevant because meaningful comparisons require standardized definitions and data fields (6). Linked datasets spanning on-site encounter, EMS transport, ED care, admission, and discharge would allow planners to distinguish true hospital burden from simple transfer counts.

Taken together, the evidence supports an integrated preparedness model in which event-health capability, EMS deployment, hospital readiness, and information systems are planned jointly and evaluated with shared metrics. The evidence is strongest for describing workload patterns and intervention configurations; it is weaker for causal claims about which individual intervention improves clinical outcomes. This distinction is important for policy: resources should be selected through risk assessment, local feasibility, and outcome monitoring rather than by assuming that the most frequently reported intervention is necessarily the most effective.

### 4.1 Limitations

This review has several limitations. First, it is a structured narrative review and therefore does not provide the exhaustive, prospectively documented screening process of a systematic or scoping review; source selection remains vulnerable to author judgment and selection bias despite a structured search. Second, the English-language restriction may underrepresent evidence from countries that host large religious, cultural, or political gatherings. Third, reliance on published literature creates potential publication bias, and the evidence base contains many retrospective, single-event, or case-study designs. Fourth, secondary reviews overlap in their underlying primary studies, so their study counts and percentages must not be summed across reviews; this manuscript therefore avoids calculating a combined denominator. Fifth, PPR, TTHR, attendance estimates, case definitions, event duration, and follow-up windows vary substantially, limiting direct comparison even when nominal units appear similar. Sixth, much of the quantitative literature concerns planned sporting or entertainment events in higher-resource settings, restricting generalizability to spontaneous gatherings, rural/remote events, Hajj-scale pilgrimages, and lower-resource health systems. Seventh, causal inference is limited: associations between on-site capability and transport or ED use may be confounded by event design, staffing thresholds, local EMS protocols, and attendee behavior. Eighth, simulation studies can overstate real-world performance because their outcomes depend on model assumptions and infrastructure availability. Ninth, relatively few studies link on-site, ambulance, ED, inpatient, and discharge data at patient level, so system-wide outcomes remain incompletely characterized. Finally, the focused September 2026 update was not a new full systematic search of all original databases and may have missed recently published evidence outside PubMed or not yet indexed.

### 4.2 Implications for practice

Preparedness should begin with a joint event-health and receiving-system risk assessment. Planners should explicitly define PPR and TTHR denominators; match staff, supplies, and treatment space to expected case mix and transport time; establish command, communications, and patient-tracking arrangements across event services, municipal EMS, and hospitals; predefine destination and escalation protocols; and monitor ED and bed-capacity effects during and after the event. High-risk or remote events may justify broader on-site capability, but the model should be evaluated locally rather than assumed to work because it was used elsewhere. Technology should be treated as an enabling system only when interoperability, redundancy, cybersecurity, connectivity, and staff training are addressed.

### 4.3 Future research

Future studies should prioritize prospective multicenter designs and linked patient-level datasets that follow individuals from the event site through EMS, ED, admission, and discharge. Core outcome definitions and denominator conventions should be standardized internationally. Comparative evaluations should examine the incremental value and cost-effectiveness of specific staffing and on-site capability models while adjusting for event risk and local system context. Digital triage and information-sharing tools require pragmatic real-world validation rather than simulation alone. Greater evidence is also needed from spontaneous gatherings, religious pilgrimages, remote/rural settings, and low- and middle-income health systems. Finally, researchers should report absolute numbers alongside percentages and clearly distinguish descriptive frequency, association, and causal effect.

## 5 Conclusion

Mass-gathering preparedness is best understood as coordination across a care continuum rather than as separate event-site and hospital plans. Published evidence shows frequent use of medical staffing, medication/procedural capability, mobile or temporary treatment space, and health-support systems, but the frequency with which an intervention is reported should not be equated with effectiveness. PPR, TTHR, EMS workload, and ED impact vary substantially by event and denominator, and hospital demand can decrease or increase depending on context and timing. The most defensible priorities are standardized reporting, linked prehospital-hospital data, explicit interagency coordination, context-specific resource design, and prospective evaluation of clinical and system outcomes.
